# The role of autoantibodies in the pathophysiology of rheumatoid arthritis

**DOI:** 10.1007/s00281-017-0627-z

**Published:** 2017-04-27

**Authors:** V. F. A. M. Derksen, T. W. J. Huizinga, D. van der Woude

**Affiliations:** Department of Rheumatology, Leiden University Medical Center, C1-R-041, Albinusdreef 2, PO Box 9600, 2300 RC Leiden, the Netherlands

**Keywords:** Rheumatoid arthritis, Autoantibodies, ACPA, Pathophysiology

## Abstract

Rheumatoid arthritis (RA) is an autoimmune disease characterized by joint inflammation. The presence of autoantibodies in the sera of RA patients has provided many clues to the underlying disease pathophysiology. Based on the presence of several autoantibodies like rheumatoid factor (RF), anti-citrullinated protein antibodies (ACPA), anti-carbamylated protein antibodies (anti-CarP), and more recently anti-acetylated protein antibodies RA can be subdivided into seropositive and seronegative disease. The formation of these autoantibodies is associated with both genetic and environmental risk factors for RA, like specific human leukocyte antigen (HLA) alleles and smoking. Autoantibodies can be detected many years before disease onset in a subset of patients, suggesting a sequence of events in which the first autoantibodies develop in predisposed hosts, before an inflammatory response ensues leading to clinically apparent arthritis. Research on the characteristics and effector functions of these autoantibodies might provide more insight in pathophysiological processes underlying arthritis in RA. Recent data suggests that ACPA might play a role in perpetuating inflammation once it has developed. Furthermore, pathophysiological mechanisms have been discovered supporting a direct link between the presence of ACPA and both bone erosions and pain in RA patients. In conclusion, investigating the possible pathogenic potential of autoantibodies might lead to improved understanding of the underlying pathophysiological processes in rheumatoid arthritis.

Rheumatoid arthritis (RA) is a chronic autoimmune disease primarily affecting the joints. RA is a heterogeneous disease that encompasses several disease subsets with probable differences in underlying pathophysiology. Via a final common inflammatory pathway, these different pathophysiological pathways might lead to a similar clinical presentation of arthritis. The best-known subdivision in RA is between ACPA-positive and ACPA-negative disease, which differ in both risk factors and clinical outcomes [1]. This review focuses on the role of autoantibodies in the pathophysiology of RA. First, the relation between autoantibodies and known risk factors for RA will be discussed. Thereafter, the specific characteristics of the autoantibody response and the pathogenic potential of the different autoantibodies are reviewed.

Several autoantibodies can be detected in serum of RA patients, of which rheumatoid factor (RF) and anti-citrullinated protein antibodies (ACPA) are the most prominent. More recently, antibodies against additional posttranslationally modified proteins were discovered, such as anti-carbamylated protein antibodies (anti-CarP) [2] and anti-acetylated protein antibodies [3]. In recent years, most research on the role of autoantibodies in disease pathophysiology has focused on ACPA, which are directed against citrullinated proteins. Citrullination is a reaction mediated by peptidyl-arginine deiminase (PAD) enzymes, which convert the DNA-encoded amino acid arginine into citrulline. This posttranslational modification occurs under both physiological and pathological circumstances. Multiple known risk factors for RA are hypothesized to be related to the development of the immune response against citrullinated proteins and thus ACPA formation. In the next section, hypotheses linking RA risk factors to autoantibody formation are discussed. Subsequently, the specific characteristics of the different autoantibodies responses are elaborated.

## Risk factors

### Genetic risk factors

Rheumatoid arthritis affects approximately 0.5 to 1% of the population. Ample research has been performed on risk factors for this disease, since it is hoped that this might provide more insight into the involved inflammatory processes and possible opportunities for prevention or treatment of RA. Several environmental and genetic risk factors increasing disease susceptibility have been identified. Twin studies have shown that genetic variation accounts for 50 to 60% of the risk on RA development [4]. The HLA-DRB1*01, *04, and *10 alleles are the strongest genetic risk factor for RA development, in particular for ACPA-positive RA [5]. Most HLA-DRB1 alleles associated with RA share an identical amino acid sequence in the peptide-binding groove, which has been termed the shared epitope (SE) [6]. The similarity in sequence has led to the hypothesis that all predisposing HLA molecules containing the SE sequence might present specific “arthritogenic” peptides, which could lead to a joint-specific autoimmune reaction. Given the strong association with ACPA-positive RA, it has been postulated that peptides presented by SE-containing alleles might be citrullinated. It was indeed shown that conversion of an arginine into a citrulline at the peptide-SE interaction site significantly increased the affinity of the peptide for the MHC molecule [7]. Furthermore, a study focusing on the crystal structure of the HLA-DRB1 antigen complex found that SE alleles preferentially bound citrullinated peptides, whereas other alleles bound both citrulline and arginine [8]. The high affinity of SE for citrullinated peptides could increase the amount of HLA peptide complexes on the surface of antigen-presenting cells (APCs), thus leading to a (possible joint-specific) T cell response [7]. However, it has proven difficult to identify the exact peptide-binding motifs for these SE molecules. Therefore, the exact peptides bound by HLA-SE molecules in vivo remain subject to further investigation.

Other theories on the role of the SE in RA development have also been postulated, since SE alleles also have another function as a ligand for cell surface calreticulin (CRT), an innate immune receptor present on most human cells and specifically on dendritic cells. The SE-CRT interaction, which is more potent when CRT is citrullinated, is able to initiate a signal transduction cascade changing the phenotype of the dendritic cell and thereby leading to skewing of T cell responses to the T helper 17 (Th17) subset and reduced regulatory T cell formation [9, 10]. However, the exact role of SE-CRT interaction to RA pathogenesis needs to be further investigated. The different hypotheses on the function of SE in RA are not mutually exclusive, and their relative importance remains unclear.

Besides the HLA region, multiple single nucleotide polymorphisms (SNP) are associated with rheumatoid arthritis [11]. Among these loci is the PTPN22 gene, the second most potent genetic risk factor for RA development [12]. PTPN22 encodes a protein tyrosine phosphatase (PTPs), which is involved in T cell and B cell antigen receptor (TCR) signaling. Thus, it may not be surprising that this gene is associated with multiple autoimmune diseases [13]. Recently, the PTPN22 risk allele was also linked to hypercitrullination of peripheral blood mononuclear cells (PBMCs), a process mediated by PAD enzymes [14]. The relationship between PTPN22 and both hypercitrullination and T and B cell receptor signaling offers new research opportunities to gain more insight in the complex events taking place during the preclinical phase of RA.

### Smoking

Of the environmental risk factors for (ACPA-positive) RA, smoking is the most important. Several theories exist on how smoking might predispose to RA. Smoking leads to higher expression of the PAD2 enzyme, increasing the level of citrullination in the lung [15]. However, it is still unclear how tolerance against citrullinated proteins is broken and ACPAs are produced, since citrullination also occurs in physiological conditions. (Hyper) citrullination alone is therefore not enough to cause a break of tolerance and leads to autoimmunity. A gene-environment interaction has been reported between HLA-DR SE alleles, and, to a lesser extent, PTPN22 and smoking. This interaction suggests interplay between T cells (on which these genetic risk factors exert their effect) and the abundance of citrullinated antigens (influenced by smoking), leading to a break of tolerance [16, 17]. Citrulline-specific T cells have been described in both SE-positive healthy individuals and in RA patients. However, the immune response in RA patients was more pro-inflammatory with a significantly higher amount of cells and skewing to a T helper 1 (Th1) memory phenotype [18, 19].

### The microbiome

Recently, the microbiome has received much attention as a possible important player in the pathophysiology of a wide variety of diseases. Also, in rheumatoid arthritis, a role for the oral and gut microbiome has been indicated. RA patients can be distinguished from healthy controls based on alterations and dysbiosis of the microbiome, for example, regarding clostridium, lactobacillus, and bifidobacteria species in the gut microbiota [20]. Alterations in the microbiome have not been found to induce arthritis, but could worsen or alleviate arthritis. It has been hypothesized that dysbiosis of the microbiome could lead to local inflammation, loss of barrier function, and bacterial translocation from mucosa to the bloodstream. Some bacterial cell wall components might molecularly mimic human autoantigens, triggering an immune response also directed against the joint [20, 21].

In this light, the epidemiological association between rheumatoid arthritis and periodontitis, a bacterial-induced chronic inflammation of the gums, is intriguing. The bacterium *Porphyromonas gingivalis*, causing severe periodontitis, might provide a pathophysiological explanation for this epidemiological relation, since ACPAs can bind citrullinated alpha-enolase of *P. gingivalis* [22]. Furthermore, this microorganism expresses a PAD enzyme, providing a source of citrullinated antigens in a pro-inflammatory environment. The proteins citrullinated by bacterial PAD might evoke an immune response that is cross-reactive with self-peptides, thus causing a break of tolerance [23]. However, mammalian calcium-dependent PAD enzymes citrullinate specific arginine residues within polypeptide chains (endocitrullination), while the PAD enzyme of P. gingivalis modifies only C-terminal arginines [24]. Therefore, antigens citrullinated by P. gingivalis PAD differ significantly from citrullinated self-antigens, which renders this molecular mimicry theory subject to debate.

New insights into this matter were provided by a recent study, which found citrullinated peptides showing endocitrullination in gingival crevicular fluid (GCF) of patients with periodontal disease. Only a single pathogen related to periodontitis, *Aggregatibacter actinomycetemcomitans* (Aa), has the potential to dysregulate citrullination by human PAD enzymes, leading to endocitrullination. Pore-forming toxin leukotoxin A (LtxA), produced by Aa, mediates this process by binding to β2 integrin (CD18) on neutrophils, leading to an influx of extracellular calcium and hypercitrullination of intracellular proteins by the cells’ own calcium-dependent PAD enzymes. In RA patients, the presence of anti-LtxA antibodies was significantly associated with both ACPA and RF positivity. Furthermore, the association between HLA-SE alleles and ACPA was exclusively found in the concomitant presence of anti-LtxA antibodies, supporting a role for LtxA and Aa in disease development. This theory on Aa-mediated hypercitrullination in human cells poses a new interesting mechanism for the generation of citrullinated autoantigens independent of molecular mimicry or citrullination by bacterial PAD enzymes [24]. Although this theory may provide evidence for the generation of autoantibodies, it does not provide a theoretical framework how antibodies induce arthritis.

## Autoantibodies in RA

It is estimated that 50–80% of RA patients harbor autoantibodies [1]. As described above, the presence of autoantibodies has allowed the identification of subgroups of RA patients that are not only more homogenous with regard to risk factors but also regarding the clinical disease course. RF, an autoantibody directed against the Fc part of human IgG, was the first autoantibody system to be described in RA. The presence of RF was considered so characteristic for RA that it was included in the 1987 ACR classification criteria for RA, despite its suboptimal specificity. Several decades later the more RA-specific ACPA were discovered [25, 26]. In the ACR-EULAR 2010 classification criteria for RA, both RF and ACPA have been included. More recently, antibodies against other posttranslationally modified proteins, i.e., carbamylated [2] and acetylated proteins were identified [3]. Seropositive RA is associated with increased radiographic progression and joint damage [27], while seronegative RA patients have higher inflammation parameters at presentation [28]. Furthermore, not only positivity for a single autoantibody but rather the conjoined presence of multiple autoantibodies might be relevant for characterizing distinct phenotypes of RA patients [29]. Autoantibodies not merely provide useful information on disease outcome but also offer insights into the development of RA. Research on the different autoantibodies and their characteristics has led to better understanding of the underlying pathophysiological processes in rheumatoid arthritis.

### Anti-citrullinated protein antibodies

As described earlier, citrullinated peptides are generated in response to a posttranslational modification mediated by PAD enzymes. Multiple antibody isotypes including IgG, IgA, and IgM directed against these citrullinated peptides are detected in RA [30]. The presence of ACPA IgA is in line with the hypothesis that ACPA is related to smoking or microbiome dysbiosis, as IgA is related to a mucosal origin of the immune response. Synovial fluid from inflamed RA joints contains citrullinated proteins, suggesting that ACPA could bind to these antigens in the joint and possibly increase local inflammation [31]. A putative target protein of ACPA is vimentin. In collagen-induced arthritis (CIA), mouse models passive transfer of ACPA cannot cause synovitis, although it can worsen preexistent synovitis [32]. Therefore, it is suggested that multiple “hits” are necessary for the development of RA. A hypothesis is that autoantibodies might specifically lead to non-resolving and chronicity of a normally temporary immune response, for example, after trauma or infection.

How might ACPA lead to inflammation? This could be mediated via binding to Fc receptors or complement activation, which is described in more detail below. Furthermore, to answer this question, the molecular structure of ACPA and specifically their glycosylation has been studied in depth over the past years. Autoantibodies in general are glycoproteins, meaning carbohydrate chains are attached to both the Fc and the Fab region of the antibody, which is essential for immune effector functions. The Fc region of ACPA has a lower level of galactosylation and sialylation compared to IgG antibodies against recall antigens [33]. Less sialylation of antibodies in immune complexes can drive osteoclastogenesis in vitro and in vivo through altered FcγR signaling. Moreover, RA patients with low levels of ACPA-IgG Fc sialylation had lower bone volumes and trabecula numbers [34]. Thus, the specific Fc glycan signature of ACPA could influence their ability to contribute to disease pathophysiology.

Strikingly, the glycosylation of ACPA is not only distinct from other antibodies in the Fc part but ACPA also more frequently have N-linked glycans in their variable domains. The prevalence of these glycans is markedly increased and they also differ in structure, with Fab glycans of ACPA having more galactose, sialic acid, and fucose residues compared to control IgG [35]. The high galactosylation and sialylation levels of the Fab-linked glycans are in marked contrast to the lower level of galactosylation and sialylation detected in the Fc part of ACPA-IgG. It remains unclear how these distinct ACPA glycosylation patterns arise, but exposure of the B cell to environmental factors such as cytokines is likely to be of importance. The influence of increased Fab glycosylation on ACPA effector functions remains an area of further investigation, but it is speculated that Fab glycosylation might affect the antibody half-life time or antibody-antigen binding [35].

### Anti-carbamylated protein antibodies

Anti-carbamylated protein (anti-CarP) antibodies also belong to the group of anti-posttranslationally modified protein antibodies (AMPA) that have been described in RA. Carbamylation is a chemical reaction mediated by cyanide in which a lysine is converted into a homocitrulline. Certain conditions, for example, renal disease, smoking, and inflammation can increase cyanide levels and thus carbamylation [36]. Similar to citrullination, increased carbamylation alone does not seem to be sufficient to break tolerance and induce autoimmunity. Only 12% of patients with renal disease harbor anti-CarP antibodies compared to approximately 44% of RA patients [37]. Smoking might contribute to the break of tolerance as a recent study showed that smoking broadened the immune response against carbamylated vimentin in mice models, but epidemiological research failed to show an association between anti-CarP and smoking in RA patients [38, 39]. The importance of smoking and other (environmental or genetic) factors necessary to break tolerance against carbamylated proteins such a fibrinogen remains to be elucidated. Although the molecular structures of homocitrulline and citrulline are very alike, ACPA and anti-CarP are distinct autoantibody classes, with anti-CarP being present in both ACPA-positive and ACPA-negative patients [2]. Moreover, anti-CarP is associated with radiographic progression in patients negative for RF and ACPA. Diagnostic classification of RA patients did not improve by adding anti-CarP testing, as RF and ACPA are already good predictors for disease [40]. Assays to test for the presence of anti-CarP most often use fetal calf serum (FCS), containing a mixture of carbamylated proteins. The exact autoantigens that anti-CarP bind in vivo remains unclear.

### Anti-acetylated protein antibodies

The latest addition to AMPAs in RA patients is anti-acetylated protein antibodies which have been described in approximately 40% of RA patients, mainly in the ACPA-positive group. Detection rates in seronegative RA patients were comparable to patients with resolving arthritis, rendering it unlikely that these antibodies will be a new biomarker helpful for diagnosing RA [3]. However, anti-acetylated protein antibodies might provide useful new insights in pathophysiology, especially in the era in which the microbiome seems to become increasingly important. Acetylation is an enzymatic process, which can be affected by bacteria, although the underlying mechanism is unclear. Therefore, anti-acetylated antibodies could provide a possible new link between microbiome dysbiosis and the development of autoimmunity in RA [3, 41].

### Development of the autoantibody response over time

The presence of the different autoantibodies in serum of (future) RA patients can be detected years before actual disease onset [42–44]. Most research on the details of the autoantibodies response prior to clinical disease manifestation is done on ACPA. From all RA patients, 50% presents with ACPA. About 4 years before disease onset, 50% of the patients that will be ACPA positive will harbor ACPA. At that time point, the ACPA levels start to increase. The profile of citrullinated antigens recognized expands extensively and isotype switching occurs. These events predict RA development in patients with undifferentiated arthritis and correlate with a rise in inflammatory cytokine levels [45–49]. Also, the Fc glycosylation pattern changes before disease onset. Galactosylation decreases while fucosylation increases, leading towards a more pro-inflammatory phenotype of the antibodies [50].

There is one feature of ACPA, which strikingly differs from the normal development of antibody responses. In general, during B cell maturation, class switching, somatic hypermutation, and affinity maturation are physiological processes occurring in germinal centers. B cells producing antibodies with sufficient avidity will proliferate, improving the efficacy of the immune response. In contrast to an immune response against recall antigens, the avidity maturation of ACPA is very limited, resulting in low avidity antibodies [51]. This is interesting as it suggests that isotype switching and avidity maturation of ACPA are relatively uncoupled. Within ACPA-positive patients, those with lower avidity, ACPA have increased joint destruction compared to patients with higher avidity ACPA, which might be mediated via a higher potency to activate complement [52].

After the disease has become clinically apparent, the ACPA profile and phenotype remains stable over time [45, 49]. The overall development of the autoantibody response over time raises many questions: it is for example thus far unclear which factors are involved in the maturation of the response before disease onset and what the role of this maturation is in disease onset.

## Pathogenic potential of autoantibodies

**Fig. 1:**
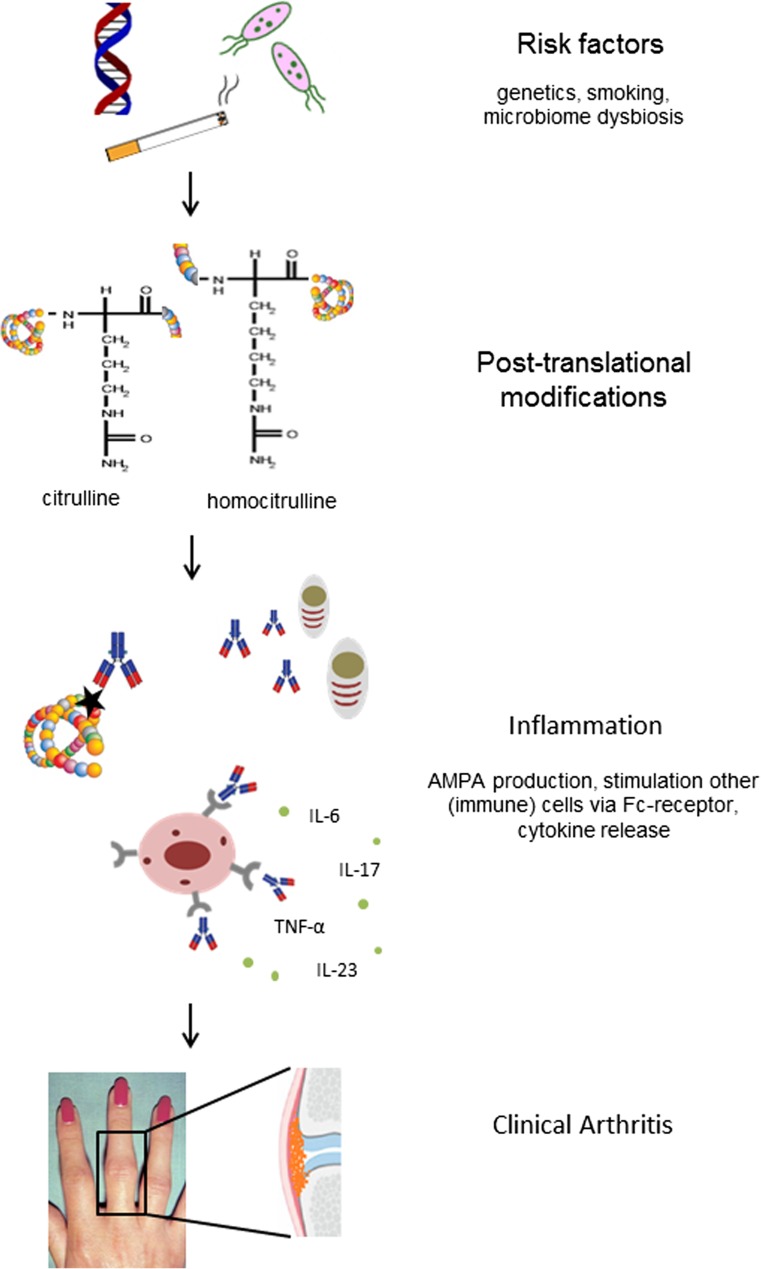
Model of the possible role of autoantibodies in disease pathophysiology. Genetic and environmental risk factors might lead to increased rate of posttranslation modification (hypercitrullination, hypercarbamylation). Autoantibodies against these posttranslational modifications are produced (ao ACPA) which can activate other (immune) cells via Fc receptors and stimulate cytokine production. The underlying inflammatory cascade eventually results in clinically apparent arthritis. *AMPA* anti-modified protein antibodies

Several features of the anti-citrulline immune response, such as the expansion of the ACPA repertoire before disease onset and the association of autoantibodies with radiographic progression, suggest a possible role in disease pathology. In addition, B cell depletion with rituximab is effective in RA patients, to a greater extent in ACPA- and RF-positive cases, arguing in favor of a role of B cells (and perhaps the autoantibodies they produce) in disease pathogenesis [53]. There are thus several lines of evidence that autoantibodies play a pathogenic role in RA. In the next section, several hypotheses are discussed regarding mechanisms by which autoantibodies might lead to ongoing inflammation and RA symptoms. In Fig. 1, the model on the possible role of autoantibodies in disease pathophysiology of RA, as discussed in this review, is depicted.

### Binding to Fc receptors

In general, antibodies exert an effect on other cells via Fc receptor binding. Similar to recall antigen immune complexes (ICs), immune complexes containing ACPA and citrullinated fibrinogen are able to stimulate TNF secretion via stimulation of Fcγ receptors on macrophages [54, 55]. The effector functions of the ACPA ICs can be modified by the presence of RF-IgM or RF-IgA, which boosts the Fcγ receptor-mediated immune response and increases complement activation [56]. This suggests that there may be a synergetic role of ACPA and RF in RA pathophysiology, which is supported by epidemiological studies showing that the combination of ACPA and RF is associated with higher disease activity [57].

### Complement activation

Another main effector function of antibodies is complement activation. The complement system can be activated via three pathways: the classical pathway (initiated by C1q), the alternative pathway (initiated by C3), and the lectin pathway (initiated by mannose-binding lectin (MBL)), which all lead to opsonisation, formation of the membrane attack complex and chemotaxis. In synovial fluid of RA patients, complement levels are reduced, while complement cleavage products are increased, indicating enhanced complement activation. It has been shown that ACPA have the ability to recruit complement via both the classical and alternative pathways, but not via the lectin pathway [58]. Taken together, the evidence suggests that ACPA have the potential to augment the immune response in RA by both Fcγ receptor binding and complement activation.

### Neutrophil extracellular trap formation in RA

The augmented generation of neutrophil extracellular traps (NETs) is another manner in which ACPA and/or RF antibodies could affect disease development or persistence. NETs are composed of highly condensed chromatin and are expelled by neutrophils to trap and kill pathogens. NETosis by both circulating and synovial fluid neutrophils is enhanced in RA patients compared to healthy controls. The relation between NETosis and autoantibodies is interesting, as NETosis exposes autoantigens, i.e., citrullinated self-proteins, in a pro-inflammatory environment [59]. Therefore, amplified citrullinated autoantigen exposure via NETosis might be involved in promoting autoantibody generation and production in predisposed hosts. It was shown that citrullinated peptides derived from deiminated histones, a component of NETs, are targeted by ACPAs [60]. B cells differentiated from synovial ectopic lymphoid tissue were reactive against the citrullinated histones exposed during NETosis [61]. On the other hand, ACPA can stimulate NETosis. NETs trigger inflammatory responses in synovial fibroblasts, including induction of IL-6, IL-8, chemokines, and adhesion molecules. ACPA might thus enhance local inflammation by increasing NETosis. Taken together, aberrant NETosis might fuel the ongoing ACPA response and leads to a circular non-resolving pro-inflammatory immune response, by which RA is characterized [59].

### Bone erosion: osteoclast activation

Seropositive RA is associated with increased damage to joints, which is commonly measured as radiographic progression [62, 63]. It has been proposed that ACPA may directly affect osteoclasts and thereby lead to the formation of bone erosions. ACPA have indeed been described to bind to the osteoclast surface and enhance differentiation of osteoclast precursors in vitro and in vivo. Adoptive transfer of ACPA has also led to increased bone resorption in mice models, although this process seemed to differ from the joint damage in RA [64]. Recently, a mediating effect of IL-8 in the relation between ACPA, stimulation of osteoclasts, and bone erosions was proposed. The finding that an IL-8 antagonist could prevent bone loss in vitro in humans and in vivo in mice supports the hypothesis that ACPA is directly linked to formation of bone erosions via IL-8 induction [65]. This hypothesis about ACPA and bone destruction is supported by preliminary data, but many questions, for example, regarding the epitopes on osteoclasts that ACPA bind to and the fine specificities of ACPA mediating the effect on bone erosions, have yet to be answered. It will be intriguing to see how this field will further develop.

### Pain

Not only radiographic progression but also chronic pain is a significant clinical problem in RA. It was investigated whether autoantibodies might also (partly) provoke these symptoms. Mouse models injected with ACPAs were found to display lasting pain-like behavior, while no sign of inflammation was present. The injected ACPAs bound surface epitopes on osteoclasts and osteoclast precursors in mouse bone marrow, subchondral bone, and epiphyseal plate. These activated osteoclasts express CXCL1 (the mouse analogue of IL-8 described earlier), which might mediate the effect on nociception [66]. This research proposes a novel theory that the presence of autoantibodies might directly contribute to arthralgia, a symptom often present before arthritis in RA.

## Final common inflammatory pathway

Described above are various scenarios about how autoantibodies could be involved in the pathogenesis of RA, but it has to be kept in mind that there is no definitive proof that these autoantibodies are in fact pathogenic. In the development of RA, a common inflammatory pathway seems to exist leading to a similar clinical presentation in patients with and without autoantibodies.

In this paragraph, a very brief overview of this final common pathway is given, in which many cell types and processes are involved. Early in the development of arthritis, activation of the innate immune system leads to an influx of leukocytes into the normally sparsely populated synovial compartment via the local expression of adhesion molecules and chemokines [67]. Less complement factors and more complement cleavage products are found locally, indicating increased complement use [68]. Also, the adaptive immune system is triggered and stimulated dendritic cells as well as costimulatory molecules are found. These circumstances facilitate activation of Th1 T helper cells [67]. More recently, the Th17 T helper cell phenotype has also been implicated as a key driver of synovitis, as these cells are potent producers of pro-inflammatory cytokines like IL-17A, IL-21, and TNF-α [69]. The pro-inflammatory T cells, cytokines, and immune complexes stimulate macrophages and fibroblast-like synoviocytes (FLS) to produce pro-inflammatory cytokines, like TNF-α, IL-1, IL-6, IL-15, and IL-23. In the RA joint, FLS have proliferated and adapted a pro-inflammatory phenotype with increased expression of chemokines, adhesion molecules, and matrix metalloproteinases (MMP). MMP can lead directly to cartilage destruction and chronic synovial inflammation. Cartilage can also be affected indirectly via cytokines leading to chondrocyte activation and tissue catabolism [70]. Besides cartilage, also bone is subject to destruction in the joint of RA patients. Activated fibroblasts, T cells, B cells, and macrophages can upregulate expression of RANK ligand, leading to osteoclastogenesis and thus bone destruction [71]. The central role of cytokines in RA synovitis is further affirmed by the successful use of monoclonal antibodies directed against these cytokines, the most well-known being anti-TNFα, in the treatment of RA. However, these therapies have the major downside that they target the immune system non-specifically, increasing the risk of infections and perhaps neoplasms. Therefore, research focusing on unraveling the precise immunopathology of RA might introduce possibilities for specific targeted therapy, which could significantly improve care for RA patients.

## Conclusion

Discoveries in recent years have shed more light on RA pathophysiology. Studies investigating the role of anti-posttranslational-modified protein antibodies, especially ACPA, have led to a better understanding of underlying pathophysiological processes. Autoantibody formation is associated with both genetic and environmental risk factors for RA, like HLA-SE alleles, smoking, and microbiome dysbiosis, offering intriguing new views on the development of RA. Also, new hypotheses on the effector functions of the autoantibodies have been postulated, indicating a role for ACPA in both bone destruction and arthralgia. However, despite descriptions of effector functions of these autoantibodies, it remains unclear if they contribute directly to disease pathogenesis and joint inflammation. Furthermore, insights into the immune response underlying seronegative disease remain limited. This continues to be an interesting area for further investigation, aiming to gain more knowledge on possible therapeutic targets. In the past decade, great progress has been achieved by blocking cytokines or receptors in the final inflammatory pathway of joint inflammation. Despite the success of these new, biologic treatments, they generally do not cure the disease and currently pose a substantial financial burden. It is therefore to be hoped that new insights into pathophysiology will allow us to achieve the ultimate goal of preventing RA.
